# Ferroptosis-Related Gene-Based Prognostic Model and Immune Infiltration in Clear Cell Renal Cell Carcinoma

**DOI:** 10.3389/fgene.2021.650416

**Published:** 2021-06-09

**Authors:** Guo-Jiang Zhao, Zonglong Wu, Liyuan Ge, Feilong Yang, Kai Hong, Shudong Zhang, Lulin Ma

**Affiliations:** Department of Urology, Peking University Third Hospital, Beijing, China

**Keywords:** ferroptosis, clear cell renal cell carcinoma, risk score, The Cancer Genome Atlas, nomogram, machine learning

## Abstract

Clear cell renal cell carcinoma (ccRCC) is one of the most common tumors in the urinary system. Ferroptosis plays a vital role in ccRCC development and progression. We did an update of ferroptosis-related multigene expression signature for individualized prognosis prediction in patients with ccRCC. Differentially expressed ferroptosis-related genes in ccRCC and normal samples were screened using The Cancer Genome Atlas. Univariate and multivariate Cox regression analyses and machine learning methods were employed to identify optimal prognosis-related genes. *CARS1*, *CD44*, *FANCD2*, *HMGCR*, *NCOA4*, *SLC7A11*, and *ACACA* were selected to establish a prognostic risk score model. Gene Ontology and Kyoto Encyclopedia of Genes and Genomes pathway analyses revealed that these genes were mainly enriched in immune-related pathways; single-sample Gene Set Enrichment Analysis revealed several immune cells potentially related to ferroptosis. Kaplan–Meier survival analysis demonstrated that patients with high-risk scores had significantly poor overall survival (log-rank *P* = 7.815 × 10^–11^). The ferroptosis signature was identified as an independent prognostic factor. Finally, a prognostic nomogram, including the ferroptosis signature, age, histological grade, and stage status, was constructed. Analysis of The Cancer Genome Atlas-based calibration plots, C-index, and decision curve indicated the excellent predictive performance of the nomogram. The ferroptosis-related seven-gene risk score model is useful as a prognostic biomarker and suggests therapeutic targets for ccRCC. The prognostic nomogram may assist in individualized survival prediction and improve treatment strategies.

## Introduction

Renal cell carcinoma (RCC), which occurs in the proximal convoluted tubule, is among the top 20 most common cancers worldwide, affecting more than 400,000 individuals each year and accounting for 2.2% of malignant tumors among all new cancer cases (Bray et al., 2018). There are three main types of RCC: clear cell (ccRCC), papillary (types I and II), and chromophobe (Frew and Moch, 2015; Moch et al., 2016), with ccRCC accounting for approximately 70% of RCC cases (Frew and Moch, 2015; Moch et al., 2016). Compared with papillary RCC and chromophobe RCC, ccRCC shows the worst prognosis according to univariate analysis (Capitanio et al., 2009). This type of renal cell carcinoma is called clear cell because most of the lipids and glycogen are dissolved in conventional histological treatments and are vacuoles under a microscope. The main risk factors for RCC are tobacco smoking, overweight or obesity, and hypertension (Frew and Moch, 2015). Deletion of *PBRM1*, *SETD2*, and *BAP1* on the 3p chromosome and mutation or deletion of *VHL* are also important driving factors for ccRCC (Mitchell et al., 2018; Turajlic et al., 2018). Loss of *VHL* leads to the accumulation of HIF, which inhibits the metabolism of fatty acids in ccRCC and leads to the accumulation of lipids (Du et al., 2017). Although tumor resection in the early stage shows beneficial effects for patients, because of the lack of early symptoms, around 25–30% of patients with RCC are diagnosed in stages III and IV, making resection difficult (Keegan et al., 2012; Capitanio and Montorsi, 2016). The 5-year survival rate of patients with metastatic RCC is only approximately 20%^1^, and ccRCC is not sensitive to radiotherapy and chemotherapy. Therefore, methods for identifying key biological markers are urgently needed to facilitate the early diagnosis of ccRCC and development of new drugs for these biological markers.

Programmed cell death (PCD) is a normal physiological process occurring in cells, and ferroptosis is a type of programmed cell death. Ferroptosis, which can be blocked by iron chelators, was first described in 2012 (Dixon et al., 2012) and then defined by the Nomenclature Committee on Cell Death as a form of PCD in 2018 (Galluzzi et al., 2018). This process differs from apoptosis, necrosis, autophagy, and other forms of regulatory cell necrosis at the morphological, biological, and gene levels. Ferroptosis is induced by the accumulation of ferric ion (Hirschhorn and Stockwell, 2019). Morphologically, according to electron microscopy analysis, iron death leads to a decrease in mitochondria and increase in membrane density (Dixon et al., 2012). The small molecule RSL3 can induce cell death, which cannot be inhibited by necrosis inhibitors such as caspase inhibitors and necrostatins (Dixon et al., 2012). When the cell cystine transporter protein is inhibited (e.g., erastin), intracellular glutathione is depleted, eventually leading to inactivation of glutathione peroxidase (GPX4) and resulting in lipid peroxidation accumulation; these factors induce iron-induced cell death (Stockwell et al., 2017). Ferroptosis helps maintain cell-death-related homeostasis in normal cells and tissues and the homeostasis of some carcinoma gene pathways. In people with iron metabolism disorder, Fe^3+^ accumulates by binding to transferrin (TF) and enters the cell through transferrin receptor-mediated endosomes. Fe^3+^ is reduced to Fe^2+^ iron by STEAP3 metalloreductase in the endosome and is then released into the intracellular environment via solute carrier family 11 member 2. After entering the cytoplasm, Fe^2+^ is stored by ferritin, which is formed by ferritin light chain and ferritin heavy chain 1. Solute carrier family 40 member 1 (also known as ferroportin-1) acts as pump to transport iron ions out of the cell (Liu et al., 2020). Increasing the release of stored intracellular iron into the cytoplasm and reducing the discharge of iron ions can increase the intracellular concentration of these ions, inducing intracellular reactive oxygen species and leading to ferroptosis (Dixon et al., 2012; Bogdan et al., 2016; Hou et al., 2016). Ferroptosis is mainly regulated by two parallel pathways involved in lipid peroxidation (Wu Y. N. et al., 2020): that involving glutathione/GPX4 (Stockwell et al., 2017) and another involving ferroptosis suppressor protein 1, ubiquinone (CoQ10), and NAD(P)H (Bersuker et al., 2019; Doll et al., 2019). If either of these systems is inactivated, Reactive oxygen species (ROS), which include superoxide and hydroxyl radicals, become active. In the cancer cells, some ferroptosis key genes are altered, reducing programmed cell death (Wu G. et al., 2020). A study had shown that glutamine can inhibit oxidation in clear cell renal cell carcinoma by ferroptosis pathway (Abu Aboud et al., 2017).

In previous research, different studies from different perspectives have explored the value of prognostic markers in ccRCC, like long non-coding RNAs (Cheng et al., 2019; Yang et al., 2020), some key genes (Yang et al., 2018; Luo et al., 2019), immune-related genes (Zhang et al., 2019), etc. Some studies have focused on the relationship between ferroptosis and ccRCC (Abu Aboud et al., 2017; Wu G. et al., 2020; Mou et al., 2021; Wang et al., 2021). Wu and colleagues did a remarkable work in clinical significance of ferroptosis-related genes (FRGs) in pan cancer and conducted a new survival model based on five risk-related FRGs for ccRCC (Wu G. et al., 2020). With the development of research, more and more genes related to ferroptosis have been found. In our study, we included more FRGs and updated FRGs survival model. Furthermore, we analyzed these genes by ssGSEA to explore the role of these genes in the immune microenvironment of ccRCC first. Considering the pathological features and the metabolic characteristics of lipid and glutamine of renal clear cell carcinoma, we tried to explore the role of ferroptosis pathway in ccRCC. Therefore, we updated on measuring ferroptosis-associated biomarkers for predicting the survival of patients with ccRCC. Furthermore, in this study, we explored the relationships between ferroptosis and the immune microenvironment in patients with ccRCC. We used these hub genes to establish a nomogram for clinical use to improve individualized prognosis assessment. By building and validating the nomogram, the prognosis prediction of patients with ccRCC in the clinic can be improved.

## Materials and Methods

### Acquisition of Transcriptome and Clinical Data

We downloaded transcriptome profiles from the publicly available The Cancer Genome Atlas (TCGA) database via the GDC data portal^2^, with the HTSeq-FPKM workflow type of all available ccRCC samples compared with normal tissues. Corresponding clinical information was obtained from the GDC portal, including age, gender, tumor grade, stage, and survival outcomes (updated by September 2020). According to TCGA data access policies and publication guidelines, there was no ethical conflict to declare, and this study did not require the approval of the ethics committee.

### Differential Ferroptosis-Related Genes Analysis and Survival Analysis

We evaluated 60 ferroptosis-related genes (Chen et al., 2020; Elgendy et al., 2020). “Limma” was used to identify differentially expressed genes (DEGs) in two groups, normal samples and tumor samples, with a false discovery rate <0.05. The “org.Hs.eg.db” package was used to obtain the Entrez ID for each DEG, and significant enrichment pathways were considered only with a false discovery rate <0.05. We selected FRGs in ccRCC to assess survival outcomes by univariate Cox regression analysis, and the *P* value was shown in the plot determined by “survival” package. Both DEGs and survival-related genes between the two groups were determined with the “VennDiagram” package. Furthermore, a heatmap was drawn by “pheatmap” package, and a Forest plot was drawn by the “survival” package to show the difference between groups.

### Determination and Validation of FRGs as Independent Prognostic Factors

Least absolute shrinkage and selection operator (LASSO) analyses were performed to identify key FRGs and establish a clinical prognostic model. Kaplan–Meier survival curves were generated to assess the model based on overall survival (OS). Time-dependent receiver operating characteristic (ROC) curves were plotted to validate the FRG prognostic model. To classify the risk of FRGs in ccRCC, we used the optimal risk cutoff to set the risk score model. The risk score for each patient, with β indicating the regression coefficient, was calculated as follows: risk score = expression level of FRG_1_ × β_1_ + expression level of FRG_2_ × β_2_ +…+ expression level of FRGn × βn (Kidd et al., 2018). According to the median expression levels of prognosis-related genes, patients were divided into high- and low-risk groups. Both principal component analysis for linear data dimension reduction and visualization by t-distributed stochastic neighbor embedding plots for non-linear dimension reduction were used to distinguish high− and low−risk groups. We also tested the expression and prognostic ability of each gene in high− and low−risk groups. Our model was verified by randomly sampling 70% of the total samples as validation set, which can be put back. We attach the verification results to the Supplementary Materials.

### Molecular Mechanism and Immune Infiltrate Analysis

Gene Ontology (GO) was constructed in 2,000 as a structured standard biological model to build a standard vocabulary of knowledge about genes and their products, covering the cellular component, molecular function, and biological process^3^. The main features of KEGG are to link genes with various biochemical reactions^4^, and KEGG is a comprehensive database integrating genomic, chemical, and systematic functional information. GO and KEGG can provide rapid gene function annotation and preliminary functional analysis. Both these methods are very popular for analyzing gene function. We analyzed the functions of FRGs by Gene Ontology (GO) and Kyoto Encyclopedia of Genes and Genomes (KEGG) with “clusterProfiler” package (false discovery rate <0.05). Single-sample Gene Set Enrichment Analysis (ssGSEA) (Barbie et al., 2009) is implemented by the extended GSEA extension; ssGSEA allows the definition of an enrichment score that represents the absolute enrichment of the gene set in each sample within a given dataset. Rank normalization of gene expression values for a given sample and generating enrichment scores using the empirical cumulative distribution function (ECDF) of genes in the signature and the remaining genes. ssGSEA was used to calculate the enrichment score of each sample in different gene sets and explore the enrichment signaling pathways in the low− and high−risk groups. ssGSEA was performed to evaluate FRGs with immune activity in ccRCC based on “GSVA” package (Hänzelmann et al., 2013).

### Establishment of the Nomogram for Clinical Application

Univariate and multivariate Cox regressions were performed to determine the association between OS and clinical characteristics. According to univariate and multivariate Cox regressions, we built a nomogram (Iasonos et al., 2008) to assess the probability of 1−, 3−, and 5-year OS of patients. To assess the predictive accuracy of this model, the C-index was used to estimate the probability of agreement between the nomogram and actual results. The C-index ranged from 0.5 to 1.0, with 1.0 indicating a perfect capacity to correctly distinguish the outcome with the model and 0.5 indicating random chance. Calibration plots were used to test the discriminative ability of the nomogram. Finally, decision curve analysis was performed to evaluate the clinical benefits of the nomogram.

### Statistical Analysis

All statistical analyses were performed using R language (version 4.0.2). A two-sided *P* value of <0.05 was considered as statistically significant. The adjusted *P* value was determined by the Benjamini–Hochberg method.

## Results

### Differentially Expressed FRGs and Survival Analysis

**FIGURE 1:**
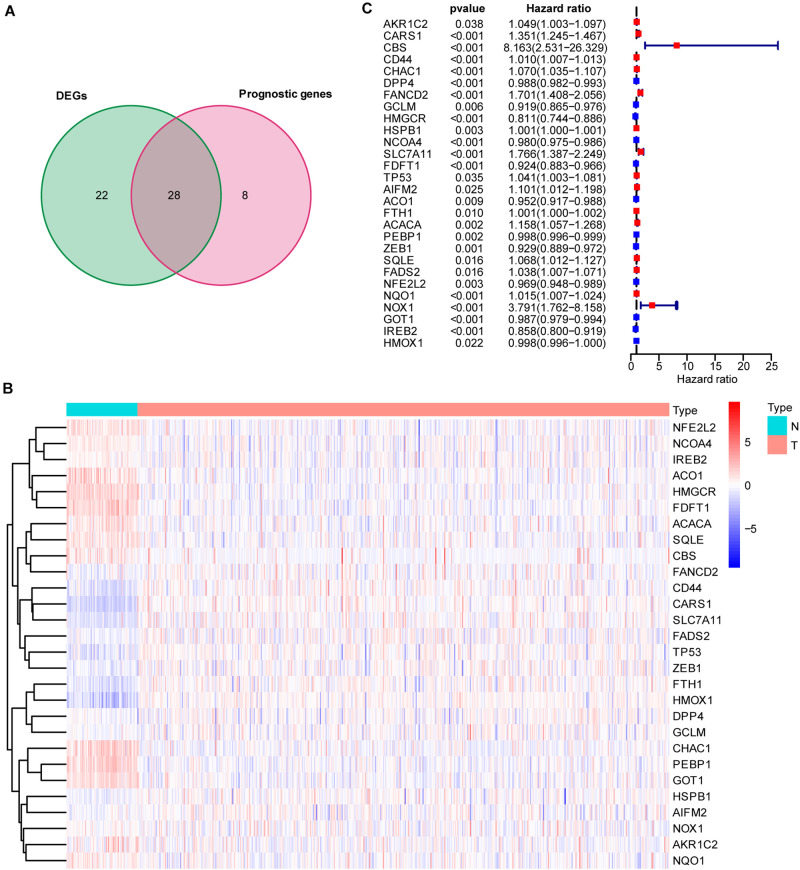
Intersection of differentially expressed ferroptosis-related genes (FRGs) and survival-associated ferroptosis-related genes. **(A)** Venn diagram showing the 28 FRGs associated with survival [intersection of the differentially expressed genes (DEGs) and prognostic genes]. **(B)** Heatmap showing differentially expressed ferroptosis-related genes between normal and tumor samples (adjusted *P* < 0.05). **(C)** Hazard ratio (HR) and 95% CI of the 28 key FRGs in univariate Cox regression. A total of 72 normal and 539 carcinoma samples were included in the analysis.

To explore the prognostic value of 60 FRGs (Stockwell et al., 2017; Chen et al., 2020) in ccRCC, 72 normal and 539 carcinoma samples were evaluated. Among them, 28 FRGs were upregulated and 22 FRGs were downregulated (adjusted *P* < 0.05). Univariate Cox regression analysis was conducted, and 36 of these 60 FRGs were found to be related to the survival of patients with ccRCC. The intersection of the FRGs based the DEGs and FRG-based survival-related genes was determined by drawing a Venn diagram (Figure 1A). Twenty-eight FRGs were selected for further analysis, with their expression displayed in a heatmap (Figure 1B). The hazard ratio, 95% confidence interval (CI), and *P* value of each key FRG are illustrated in a forest plot (Figure 1C).

### ROC Curve Indicated Good Performance for the Seven FRGs in the Risk Score Model for Predicting the OS of Patients With ccRCC

**FIGURE 2:**
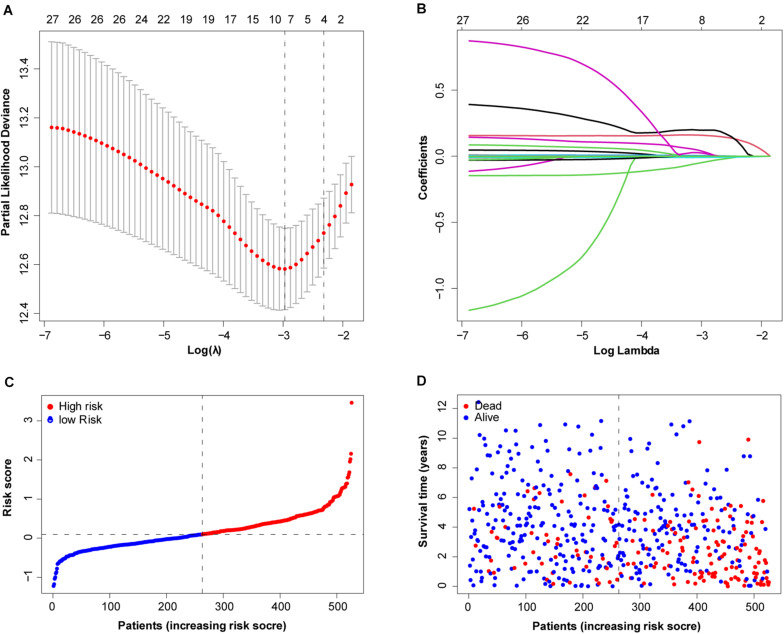
Determination of ferroptosis-related genes (FRGs) as an independent prognostic factor. **(A)** Partial likelihood deviance was plotted against log (lambda). Vertical dotted lines indicate the lambda value with minimum error. The largest lambda value is where the deviation is within one standard error (SE) of the minimum. **(B)** Least absolute shrinkage and selection operator (LASSO) coefficient profiles of FRGs and selected seven FRGs. **(C)** Risk score curve of ferroptosis signature. **(D)** Patient survival status and time distributed by FRG-based risk score.

**FIGURE 3:**
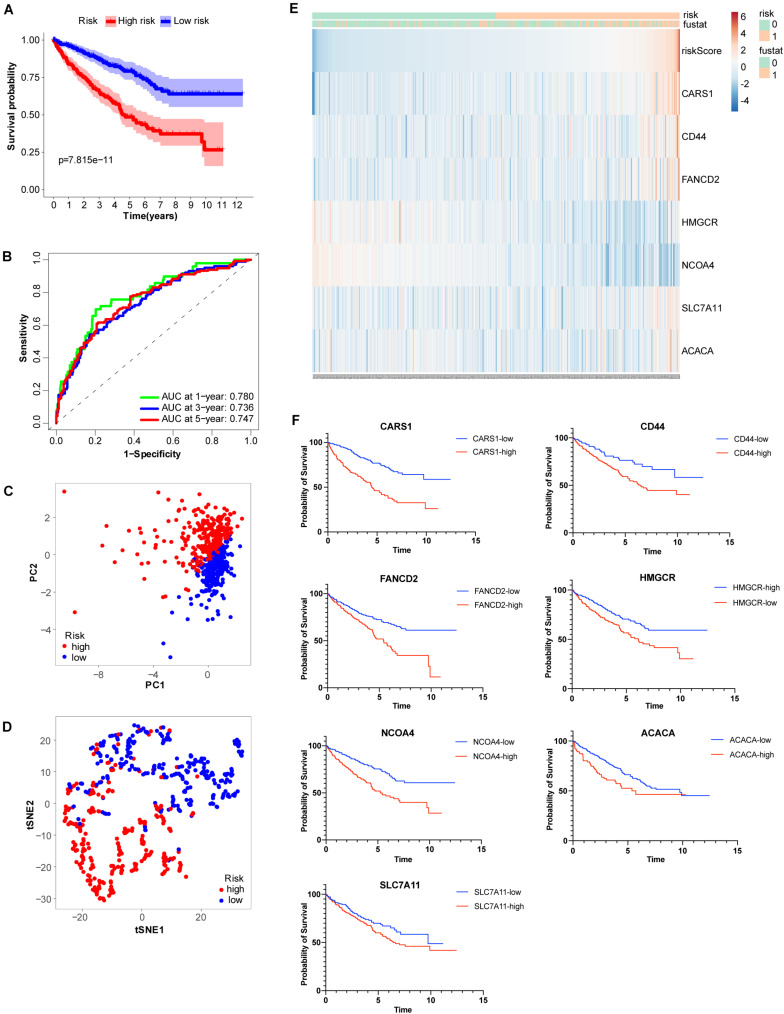
Analysis of ferroptosis-related genes (FRGs) as an independent prognostic factor. **(A)** Kaplan–Meier survival curves show overall *P* values of survival and 95% CI for high− and low−risk patients with clear cell renal cell carcinoma (ccRCC) based on the novel model. **(B)** One−, 3−, and 5-year receiver operating characteristic (ROC) curves and area under the curve (AUC) = 0.780, 0.736, and 0.747, respectively. **(C)** Principal component analysis of FRGs showed two patient clusters. **(D)** t-Distributed stochastic neighbor embedding plots for non-linear dimension divided ccRCC into two groups by the new risk score model. **(E)** Heatmap showing differentially expressed ferroptosis-related genes between high− and low−risk. **(F)** Kaplan-Meier survival curves of the seven genes in ccRCC.

To more efficiently identify hub genes, a machine learning method (LASSO) was used. Seven FRGs – cysteinyl-tRNA synthetase 1 (*CARS1*), cluster of differentiation 44 (*CD44*), Fanconi anemia complementation group D2 (*FANCD2*), 3-hydroxy-3-methylglutaryl-CoA reductase (*HMGCR*), nuclear receptor coactivator 4 (*NCOA4*), solute carrier family 7 member 11 (*SLC7A11*), and acetyl-CoA carboxylase alpha (*ACACA*) – were selected to build a ferroptosis signature model based on minimum criteria (Figures 2A,B). We also made a heatmap and a survival status plot of these seven genes (Figures 3E,F). According the median risk score, we divided patients with ccRCC into low− and high−risk groups and built a new survival model comprised of the seven FRGs (Figures 2C,D). The equation of the FRG-based prognostic signature model was as follows: risk score = (0.1581 × expression value of *CARS1*) + (0.0040 × expression value of *CD44*) + (0.1968 × expression value of *FANCD2*) + (−.0464 × expression value of *HMGCR*) + (−0.0091 × expression value of *NCOA4*) + (0.0225 × expression value of *SLC7A11*) + (0.0352 × expression value of *ACACA*). To investigate the prognostic prediction performance, Kaplan–Meier survival curves were used. Patients in the high-risk group showed significantly shorter survival rates than those in the low-risk group (Figure 3A). The heatmaps showed the difference from the high− and low−risk groups (Figure 3E). Meanwhile, the outcomes of Kaplan–Meier survival curves of the seven genes in ccRCC also showed that the low-risk group have a better prognosis result (Figure 3F). ROC curve analysis performed to determine the prognostic prediction performance of the new survival model in patients with ccRCC revealed area under the curve (AUC) scores of 0.780, 0.736, and 0.747, for 1−, 3−, and 5-year survival, respectively (Figure 3B). As a novel prognostic factor, the FRG-based risk score can effectively help to predict the survival status of patients with ccRCC. Furthermore, we applied two widely applied dimension reduction methods, principal component analysis for unsupervised learning, and linear and t-distributed stochastic neighbor embedding for non-linear dimension reduction. These two plots revealed good distinction between the high− and low−risk groups (Figures 3C,D). Validation set also showed that the low-risk group has better prognosis result than the high-risk group (*P* < 0.05, Supplementary Materials 1).

### Molecular Function Analysis Indicated the Relationships Between FRGs and Immune Infiltrates

**FIGURE 4:**
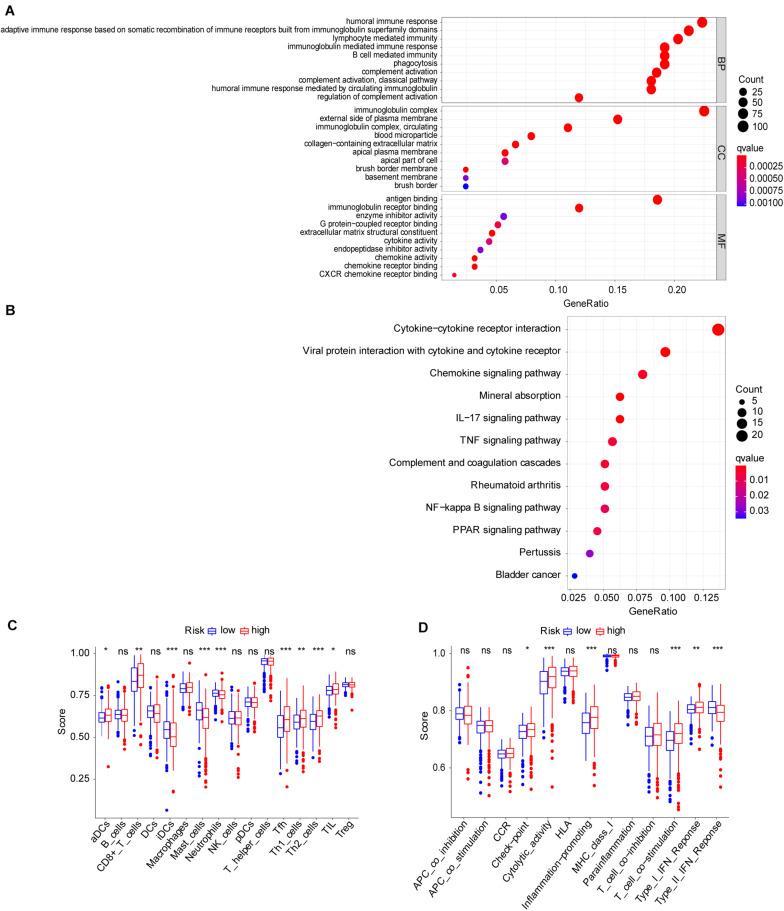
Molecular mechanism and immune infiltrate analysis of ferroptosis-related genes (FRGs) in clear cell renal cell carcinoma (ccRCC). **(A,B)** Kyoto Encyclopedia of Genes and Genomes (KEGG) pathway analysis revealed the top signaling pathways represented by the FRGs and FRG-interacting genes. **(C,D)** Single-sample Gene Set Enrichment Analysis (ssGSEA) of the seven FRGs in The Cancer Genome Atlas (TCGA) datasets. *P < 0.05, **P < 0.01, ***P < 0.001.

To explore the molecular function and potential signaling pathways related to the seven prognostic genes, we conducted GO and KEGG analyses. GO enrichment analysis (biological process, cellular component, and molecular function) of the seven FRGs revealed a significant relationship between humoral immune response, immunoglobulin complex, and antigen binding (Figure 4A). KEGG analysis showed that these genes were significantly associated with the cytokine–cytokine receptor interaction (Figure 4B). Furthermore, ssGSEA was conducted to detect potential relationships between immune-related signaling pathways and the seven prognostic genes. As shown in Figures 4C,D, among the low− and high-risk groups, significant differences (*P* < 0.001) were observed in some immune-function-related pathways such as cytolytic activity, inflammation-promoting, T-cell costimulation, and type II interferon response. The cell proportions were significantly different for lymphoid dendritic cells, mast cells, neutrophils, follicular helper T cells, and Th2 cells between the high− and low−risk groups (*P* < 0.001).

### Construction and Validation of FRG-Based Risk Score Prognostic Model

**FIGURE 5:**
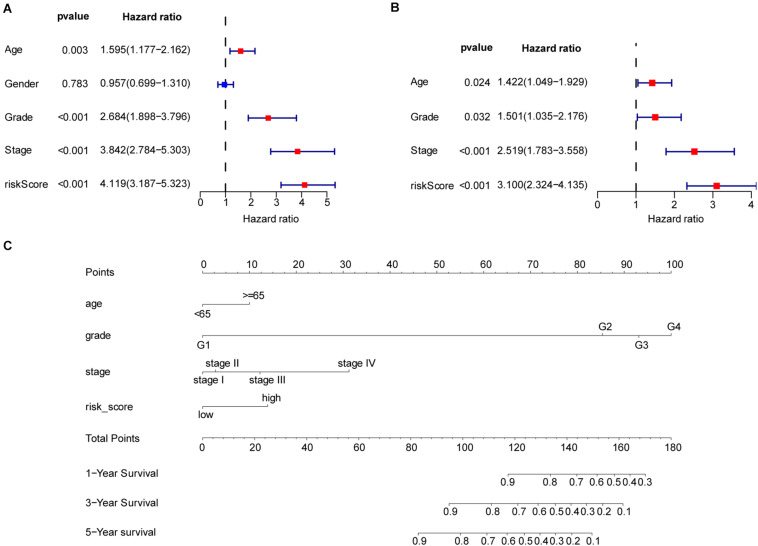
Nomogram to predict the survival probability of patients with clear cell renal cell carcinoma (ccRCC). **(A)** Univariate Cox regression of clinical characteristic and risk score model. **(B)** Based on the results of univariate Cox regression, hazard ratio (HR), and 95% CI of multivariate Cox regression analysis, age, grade, stage, and risk score showed significant differences between the high− and low-risk groups. **(C)** Prognostic nomogram for predicting the survival of patients with ccRCC. Every predict factor corresponds to a score. Clinically, the corresponding score can be obtained according to the patient’s condition, and the total score can be obtained by adding each score, which corresponds to the corresponding survival probability.

**FIGURE 6:**
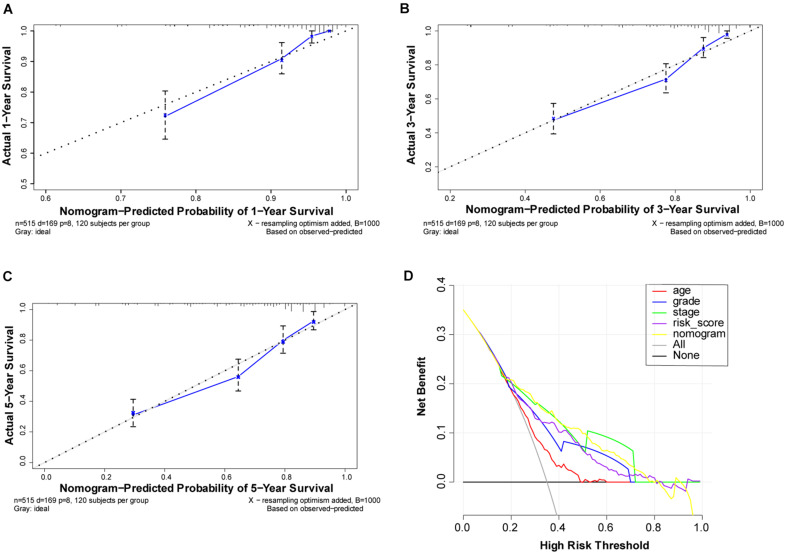
Performance of ferroptosis-related gene (FRG)-based risk score prognostic model. **(A–C)** Calibration curves of the nomogram for predicting survival at 1, 3, and 5 years. If the actual curve is closer to the ideal curve, the nomogram prediction accuracy is higher **(D)** Decision curve analysis of this nomogram. Including the risk score model, the nomogram shows that advanced age, grade, risk score, and partially were better than stage for predicting survival.

To better understand the prognostic value of the risk score prognostic model and other clinical characteristics in ccRCC, we performed both univariate and multivariate Cox regression analyses to identify factors affecting OS in TCGA dataset (Figures 5A,B). As staging is based on tumor–node–metastasis (TNM) classes, only stage was included in our analysis. Four independent prognostic factors – age, seven FRG-based risk score, grade, and stage – were included in the prediction model. We estimated the nomogram associated with FRGs to predict the OS probability at 1, 3, and 5 years and evaluated its predictive ability (Figure 5C). The C-index of the nomogram was 0.705 (95% CI, 0.663−0.747; *P* = 6.885 × 10^–21^). The calibration plots for 1−, 3−, 5-year survival showed a favorable predictive ability of the nomogram in predicting the OS of patients with ccRCC. Compared with other clinical prognostic factors, decision curve analysis showed that the nomogram was more beneficial for patients (Figure 6).

## Discussion

Renal cell carcinoma is a malignant tumor with an occult incidence and is difficult to diagnose in early stages. Although previous studies have investigated numerous molecular biomarkers and multiple gene expression signatures for ccRCC (Wu G. et al., 2020), the clinical utility patterns and immune microenvironment of FRGs have not been comprehensively analyzed. A Cox proportional-hazards model was developed and validated to predict the survival probabilities of patients, resulting in the selection of seven FRGs. These genes may play important roles in the occurrence and development of ccRCC. Based on related studies of FRGs, we explored the mechanism and immune microenvironment of ccRCC from the perspective of ferroptosis. We hypothesized that the combination of FRGs and clinical characteristics could accurately predict the prognosis of ccRCC. Thus, we constructed a risk score model based on these genes and applied it to establish a nomogram.

Ferroptosis disrupts iron and lipid metabolism, leading to the accumulation of reactive oxygen species and lipid peroxidation products. The molecular biological functions of seven FRGs were reported previously. We evaluated the roles of these genes in ccRCC; NCOA4 is a cargo receptor that is highly enriched in autophagosomes. The combination of NCOA4 and ferritin heavy chain 1 promotes ferroptosis by releasing free iron stored in ferritin heavy chain 1. Our risk score analysis showed that high expression of NCOA4 in patients has a negative risk score. Thus, NCOA4 may have some protective effects in patients. Polyunsaturated fatty acids are the main oxidants in the cytoplasm and are synthesized by acetyl-CoA. ACACA/ACC1 catalyzes the rate-limiting step of fatty acid synthesis and triggers cell death (Dixon et al., 2015). However, phosphorylation of ACACA limits its function, thus protecting cells from cell death (Lee et al., 2020). GPX4 mediates the conversion of glutathione to GSSG, which can reduce lipid peroxidation and inhibit ferroptosis. Glutathione is an intracellular antioxidant that can convert to GSSG by GPX4. As the precursor material of glutathione, cysteine can be converted from cystine that is transported into cells by the x_*c*_^–^ system; this system consists of light-chain subunit SLC7A11 and heavy-chain subunit SLC3A2. Extracellular cystine is imported with intracellular glutamate release, and SLC7A11 is specific for the x_*c*_^–^ system (Lin et al., 2020). Thus, SLC7A11 is an important transporter that maintains intracellular cystine homeostasis (Bannai, 1986). Transport of cystine inhibits peroxidation and reduces cell death. SLC7A11 can also affect the occurrence and progression of tumors in other ways (Lin et al., 2020). CD44 is a common biomarker of cancer stem cells as well as tumor cell invasion and metastasis (Xu H. et al., 2020). Moreover, CD44 acts on SLC7A1 to stabilize SLC7A11 and inhibit iron death in combination with the x_*c*_^–^ system (Ishimoto et al., 2011). The HMGCR enzyme catalyzes the rate-limiting step of mevalonate-derived terpene biosynthesis including isopentenyl pyrophosphate to promote the maturation of GPX4 (Warner et al., 2000; Viswanathan et al., 2017). Previous studies showed that an increase in GPX4 also inhibits cell death and promotes the tolerance of tumor cells to lipid peroxidation, contrasting our results. According to our risk score, high HMGCR expression, which may cause hypercholesterolemia, is a protective factor in renal ccRCC. This outcome was also observed previously (Wu G. et al., 2020). Statins can inhibit the function of HMGCR and are widely used to treat hypercholesterolemia. Statins are administered as a protective factor to patients with ccRCC (Neumann et al., 2019; Okubo et al., 2020). As no medication information was given in the clinical data, the impact of statin use on our results is unclear. CARS, the cysteinyl-tRNA synthetase, suppresses ferroptosis induced by erastin, which inhibits the cystine–glutamate antiporter known as the x_*c*_^–^ system (Hayano et al., 2016). FANCD2 is a nuclear protein that may regulate iron metabolism by ferritin heavy chain 1, TF, and lipid peroxidation by GPX4 to prevent ferroptosis (Song et al., 2016).

The cross-talk between tumor cells and non-tumor cells like immune cells, fibroblasts, and myeloid lineage cells consists the tumor microenvironment, which plays an important role in tumor occurrence and progression. The immune cells including T cells, dendritic cells, and macrophages are both prohibited and promoted tumorigenic depending on the complex cross-talk (Schulz et al., 2019; Yang et al., 2021). With tumor growth, fibroblasts and macrophages began to have affinity to tumor cells, and immune-suppressor cells, including myeloid-derived suppressor cells, and Treg cells were mobilized. As the consequence, many immune survey pathways were blocked. In ccRCC, there are abundant infiltration of immune cells, such as CD4+ T cells, CD8+ T cells, and natural killer cells (Komohara et al., 2011). However, contrary to what people expected, the number of CD4+ T cells and CD8+ T cells was negatively correlated with the survival time of patients (Nakano et al., 2001). With the further study of the interaction between tumor cells and immune cells, the prospect of effective immunotherapies for the treatment of patients with cancer is now becoming a clinical reality. The most widely studied is the expression of key receptors on the surface of T cells that prevent them from becoming activated, cytotoxic T lymphocyte antigen 4 (CTLA4), programmed cell death-1 (PD-1), and programmed death-ligand 1 (PD-L1). Based on the above two immune checkpoints, a series of monoclonal antibody drugs targeting CTLA4, PD-1, and PD-L1 have been developed, which have all been shown to prevent the interaction of these molecules with their respective inhibitory target proteins, thereby restoring antitumor immune responses (Xu W. et al., 2020).

Our study also found the association between FRGs and immunity, so we further conducted ssGSEA. ssGSEA is used to calculate enrichment scores for each paired sample and gene set and is an extension of GSEA for systematically analyzing the association of FRDs and the immune microenvironment (Barbie et al., 2009). Our results partially explain the possible reaction between the immune system and ferroptosis in ccRCC. As shown in Figure 4C, the ssGSEA score of CD8+ T cells was higher in the high-risk group, and the ferroptosis signaling pathway represented by FRGs was more active in CD8+ cells. This may imply that CD8+ T cells are more likely to die of programmed cell death. Additionally, studies suggested that T-cell activation state are strong prognostic determinants of ccRCC (Nakano et al., 2001; Adotevi et al., 2010). Interferon-γ is secreted by CD8+ T cells, leading to the downregulation of the expression of SLC7A11 of tumor cells, decline in levels of intracellular cystine, and prompting ferroptosis (Wang et al., 2019). By exporting glutamate, the x_*c*_^–^ system also affects the tumor microenvironment (Lin et al., 2020). CD44 not only affects the immune system by affecting SLC7A11 but also regulates the migration and activation of T cells in a variety of ways (Kano et al., 2014). NCOA4 can also affect the recognition of tumor cells by interfering with interferon-γ (IFN-γ) receptor signaling (Sottile et al., 2019). CARS could be secreted from cancer cells to activate immune responses via specific interactions with TLR2/6 of dendritic cells (Cho et al., 2020). Inhibition of ACACA function was reported to enhance the formation of CD4+ T memory cells by disturbing the fatty acid metabolism pathway (Endo et al., 2019). Iron death is a double-edged sword. If the iron death pathway is activated in T cells, the effect of T cells on tumor is decreased. Recent studies have shown that T cells can also induce iron death in tumor cells, thus inhibiting tumor cells (Li and Li, 2020). The function of immune cells in tumor may be related to their activation status and regulatory factors and present affinity and antitumor characteristics at the same time (Zamarron and Chen, 2011; Gajewski et al., 2013).

The ferroptosis provides a new idea for the study of tumor immune infiltration. Future research may reveal the therapeutic direction of tumor immunotherapy by clarifying the mechanism of cytokines. Based on the function of GO and KEGG, our cellular function analysis is to explore the function of these FRGs. The individual function of these genes needs to be verified and identified in further experiments.

The nomogram is based on regression analysis, which integrates multiple clinical prognostic predictors. According to the contribution of each predictor to the overall survival rate, the points are scored, and then, the total points are obtained by adding the scores. According to the total score, the 1−, 3−, or 5-year overall survival rate of patients is calculated. The nomogram makes the prognosis model more readable and convenient for clinicians to evaluate the survival prognosis of patients.

To establish the nomogram, we detected seven FRGs most closely related to the clinical prognosis of ccRCC. Patients were divided into high− and low−risk groups according to the risk score. Clinical stage was determined based on TNM classification. Therefore, to avoid duplication of cancer classification, only stage classification was considered in this study. Age, gender, histological grade, and stage were integrated into the Cox proportional hazards models to achieve better predictive performance. FRG prognostic nomograms based on the results of the Cox regression model were developed and validated to quantitatively estimate the survival probabilities of patients with ccRCC. The C-index, decision curve analysis, and calibration plots demonstrated favorable consistency between the actual and predicted survival. Thus, our gene signature and nomogram may provide an accurate and reliable prediction approach for the prognosis of patients with ccRCC and help clinicians optimize and personalize treatment strategies.

There were some limitations to our research, which will be further explored in our future work. We attempted to verify the above model by proteomics analysis but did not find all seven FRGs in the Clinical Proteomic Tumor Analysis Consortium. In some studies, ACACA was shown to promote programmed cell death, whereas other studies showed that phosphorylation of ACACA inhibits iron-related cell death (Lee et al., 2020). We hypothesized that the phosphorylation of ACACA in ccRCC could cause loss of function of this enzyme. We were unable to verify this hypothesis, as our study was performed at the RNA level. Many messenger RNA (mRNA) isomers play different roles in tumor, which could not be examined using databases. Thus, mRNA isomers were not considered in our research. Among the numerous forms of CD44 produced by alternative mRNA splicing, CD44v functions closely integrated to induce the x_*c*_^–^ system and responds to SLC7A1 (Ishimoto et al., 2011). HMGCR has been predicted to inhibit ferroptosis, which contrasts the results observed using our risk score. Whether HMGCR is affected by statin administration to patients requires further analysis. Additionally, our research and hypothesis are based on mRNA level; the functions of these seven FRGs in ccRCC should be examined in further *in vivo* and *in vitro* studies. We searched several databases but did not find other ccRCC sequencing data with similar patient baseline and sequencing platform. The testing dataset in this study should be evaluated in further research.

In conclusion, this study explored the role of FRGs in ccRCC and the relationship between FRGs and the immune environment in ccRCC. We developed an FRG-based prognostic nomogram to improve estimation of the survival rate of patients with ccRCC. This model may be used by medical professionals to develop further treatment options and perform in-depth studies of the molecular biology of ccRCC.

## Data Availability Statement

Publicly available datasets were analyzed in this study. This data can be found here: https://portal.gdc.cancer.gov/.

## Ethics Statement

Written informed consent was obtained from the individual(s) for the publication of any potentially identifiable images or data included in this article.

## Author Contributions

LM, SZ, and G-JZ designed and conducted the study. G-JZ, ZW, and FY analyzed the data. G-JZ wrote the manuscript. LM, KH, and LG reviewed the draft. All authors read and approved the final manuscript.

## Conflict of Interest

The authors declare that the research was conducted in the absence of any commercial or financial relationships that could be construed as a potential conflict of interest.

## Footnotes

1 https://seer.cancer.gov/

2 https://portal.gdc.cancer.gov/

3 http://geneontology.org/

4 https://www.kegg.jp/
