# Supplementary material for: Ferroptosis-Related Gene-Based Prognostic Model and Immune Infiltration in Clear Cell Renal Cell Carcinoma
Source: Front Genet. 2021 Jun 9;12:650416. doi: 10.3389/fgene.2021.650416 (PMC8220217; doi:10.3389/fgene.2021.650416)
Supplement: Supplementary file 1 [file Data_Sheet_1.docx]

Supplementary 1

**Figure 1**．Kaplan-Meier survival curves of validation set show overall P values of survival and 95%CI for high- and low-risk patients with ccRCC.Our risk score model was verified by randomly sampling 70% of the total samples as validation set which can be put back.
